# First Trimester Ferritin Is Superior over Soluble Transferrin Receptor and Hepcidin in Predicting Anemia in the Third Trimester: Result from a Cohort Study in Indonesia

**DOI:** 10.1155/2020/8880045

**Published:** 2020-10-08

**Authors:** Raden Tina Dewi Judistiani, Tita Husnitawati Madjid, Budi Handono, Hadyana Sukandar, Setyorini Irianti, Lani Gumilang, Sefita Aryuti Nirmala, Budi Setiabudiawan

**Affiliations:** ^1^Department of Public Health, Faculty of Medicine, Universitas Padjadjaran, Bandung, Indonesia; ^2^Post Graduate Study in Obstetrics and Gynecology Specialists, Faculty of Medicine, Universitas Padjadjaran, Dr Hasan Sadikin Hospital, Bandung, Indonesia; ^3^Bachelor in Midwifery Program, Faculty of Medicine, Universitas Padjadjaran, Bandung, Indonesia; ^4^Department of Child Health, Faculty of Medicine, Universitas Padjadjaran, Dr Hasan Sadikin Hospital, Bandung, Indonesia

## Abstract

**Introduction:**

Anemia in the third trimester has been identified as a risk factor for maternal and fetal morbidity that might lead to mortality. Due to its high cost, finding the best marker to predict anemia became more important to allow early prevention. Only one of ferritin, hepcidin, or soluble transferrin receptors can be picked for the prediction of anemia in the third trimester especially in low-resource setting.

**Objective:**

This study aimed at defining the best marker among ferritin, hepcidin, or soluble transferrin receptor (sTfR) in the first trimester for prediction of anemia in the third trimester. *Materials, Methods*, *and Setting*. This diagnostic study was nested on the cohort study of vitamin D and its impact during pregnancy in Indonesia. Singleton pregnant mothers with normal fetus were recruited in the first trimester from four cities in West Java, Indonesia. The 304 pregnant women were screened for hepcidin, ferritin, and sTfR level in the sera. All biomarkers were measured by ELISA. Complete blood count (CBC) was done by impedance method measurement (Sysmex^R^). Only subjects with complete data were included in analysis for diagnostic study to compare the three markers by finding the best receiver operating curve (RoC), likelihood ratio (LR), and risk estimate (RR).

**Result:**

One-hundred and eighty-one pregnant women were eligible for analysis. The result of this study showed that the serum ferritin level in the first trimester was the best marker to predict anemia in the third trimester of pregnancy. Hepcidin and sTfR performed poorly. A new cutoff point of ferritin level ≤27.23 ng/ml yielded the best ROC with 67% area under curve (95% CI 60%–75%, *p* < 0.0001, Youden index *J* 0.28), specificity 86.29% (95% CI 79.0%–91.8%), LR (+) 3.07 (95% CI 1.8–5.3), and RR 2.48 (95% CI 1.67–3.68). These last figures were better than the previously used cutoff point of ferritin level below 30 ng/ml.

**Conclusion:**

This study provided evidence that the serum ferritin level ≤27.23 ng/ml in the first trimester was the best marker to predict anemia in the third trimester. It was valuably useful for secondary screening of anemia in pregnancy, targeting subjects who may need rigorous approach for iron deficiency treatment in the prevention of anemia in pregnancy.

## 1. Introduction

Anemia is a global major health problem [1]. Based on surveys in South Asia around 1993–2005, anemia affected 48.5% of pregnant women, and the figured changed to 48.7% in 2011 [1, 2]. The prevalence of anemia was relatively constant despite increment in the proportion of pregnant women population being surveyed from 80% to 97.8% [1, 2].

Due to the devastating impact of anemia, especially among vulnerable subjects like pregnant women, adequate strategies in prevention, early detection, and prompt treatment are needed. A report from South Africa showed that anemia in pregnancy had deleterious effects such as abruptio placenta, preterm birth, and stillbirth [3]. Another report concluded that maternal anemia increased the risk for small for gestational babies [4]. The cohort study in which this study was nested also reported high prevalence of anemia and hypovitaminosis D and intrauterine growth restriction (10%) [5–7]. Reports stated that the prevalence of anemia increased by gestational age [5, 8]. In the pregnancy cohort of this study, anemia had 4-fold increase of prevalence in the third trimester, and relative risk (95% CI) for anemia in the third trimester among subjects with vitamin D deficiency and insufficiency in the first trimester (combined) was 2.96 (0.36–24.63) [5].

Anemia is included as a target in Sustainable Development Goals (SDGs) number 2, which were to end hunger, achieve food security, and improve nutrition [9]. Furthermore, the effort to combat anemia can be related to SDG goal number 3 that is to ensure healthy lives and promote well-being for all at all ages [9]. The goal in reduction of anemia prevalence was set at 50 percent from 40% to 20% [10].

The complexity of causal factors and the delay of detection/treatment could be the root of the problems that interventions for anemia seemed to fail; therefore, early detection is important. Most reports stated that anemia in pregnancy is mostly due to nutrient deficiency, such as iron, folic acid, vitamin A, and vitamin B12 [1, 2, 11, 12]. Other diseases such as malaria, hookworm infestations, schistosomiasis, HIV infection, and genetically inherited hemoglobinopathies should also be considered [11, 12]. Less availability and affordability of laboratory methods to determine the causes of anemia may limit opportunities to treat anemia. One also needs to consider that anemia of inflammatory condition may disguise the root cause [13].

Inflammation is part of the physiological changes of pregnancy. Some inflammation marker levels have been detected in higher level among pregnant women than in nonpregnant women, such as C-reactive protein and proinflammatory cytokines [14]. Anemia of inflammation (AI) is a common, typically normocytic normochromic anemia that is caused by an underlying inflammation, indicated by low serum iron concentrations despite adequate iron stores (ferritin) [13]. Differentiating AI from iron deficiency anemia (IDA) is difficult; furthermore, the two conditions may coexist. Traditionally, ferritin has been used as the marker for iron storage, because iron stores in the body exist primarily in the form of ferritin. One disadvantage of ferritin was that ferritin has been known as a positive acute phase response protein, so that it may not reflect the size of the iron store in inflammatory condition [15]. Adjusting thresholds of serum ferritin for iron deficiency was needed [16]. Another marker for iron metabolism in inflammation is hepcidin which has been addressed as the master of regulator of systemic iron bioavailability, including in pregnancy [8]. Hepcidin was found at lower level in nonpregnant state and that pregnant women with lower hepcidin level had higher rate of maternal-ingested transplacental iron transfer [8]. Although the hepcidin level was significantly associated with ferritin, the study by Koenig et al. did not assess their diagnostic value for the detection of anemia [8].

In a report, it was stated that soluble transferrin receptor (sTfR) concentration in serum was useful for diagnosis of iron deficiency, especially in a state when one is compromised by inflammatory condition; however, the report did not include pregnant women [17]. Another study reported that serum transferrin receptors were significantly higher in anemic pregnancies than in nonanemic pregnancies and that the rise is higher by severity of anemia [18].

From the cohort of this study, it was reported that the correlation of ferritin level and anemia in the first trimester was weak (*r* = 0.147, *p*=0.038), but no correlation was found between first trimester ferritin level and second or third trimester anemia [5]. For public health and clinical purposes, we need to use the best available evidence. Ferritin has been readily used in almost in every laboratory in big cities, but the use of hepcidin and sTFr is still limited in research field only. This diagnostic study aimed at finding the best marker in the first trimester to predict anemia in the third trimester. Our previous report showed that the proportion of anemia in the third trimester increased among pregnant women with cholecalciferol deficiency in the first trimester, but it did not show correlation with the ferritin level [5]. Three markers were picked: hepcidin, ferritin, and soluble transferrin receptor.

Sharma et al. reported that the serum ferritin level was found lower among pregnant women with anemia [18]. The cross-sectional study compared ferritin and serum transferrin receptors among pregnant women with and without anemia [18]. Achebe and Gafter-Gvili stated that iron deficiency was the only clinical situation associated with extremely low values of ferritin [19]. Previous studies were mostly in cross-sectional design and did not evaluate the diagnostic value of hepcidin or ferritin or transferrin receptor for detection of anemia in pregnancy [8, 15, 18–20]. To the best of our knowledge, until this paper was written, only three cohort studies on pregnant women in Indonesia were found in Pubmed, but none of the cohorts studied anemia in pregnancy [21–23]. The need to find the best possible marker to predict anemia is evident, so that secondary prevention of anemia progression can be managed as in the recommendations by WHO [24] and its complication can be avoided. In this study, diagnostic study was performed on ferritin, hepcidin, and sTfR in the first trimester, with additional analysis for cholecalciferol and calcitriol.

## 2. Materials and Methods

This study is a part of cohort study on vitamin D status and its impact during pregnancy and childhood in Indonesia. The cohort also studied some more nutritional aspects besides vitamin D. Submission of pregnant women in the first trimester as subjects began from late 2016, and the follow up of the offspring has been ongoing until 2020. The selection and recruitment processes of the cohort have been published previously [5]. The number of subjects was added to the previous report as the cohort progressed. Participations in the study and publications of laboratory results were based on written consent, without disclosing the identity of pregnant women.

Ten millilitres of blood was drawn from each pregnant woman on the first, second, and third trimesters; it was required for routine analysis and also for the purpose of the cohort study. Serum was separated and stored in −20°C prior to laboratory analysis. Complete blood count was calculated using the automated hematology analyzer with impedance method measurement (Sysmex XP-100, Japan). Measurements of serum ferritin, hepcidin, sTfR, cholecalciferol, and calcitriol were performed by ELISA.

Descriptive statistics were compared between subjects with and without anemia in the third trimester, which required that subjects had complete data on haemoglobin in all trimesters. Diagnostic study analysis and risk estimates were performed among subjects with complete data on hemoglobin in all trimesters and the three markers ferritin, hepcidin, and sTfR. Additional analysis was done for cholecalciferol and calcitriol in the first trimester due to new results. The correlation test between all variables was done by SPSS version 10. The receiver operating curve (ROC) and likelihood ratios analysis were performed by Med Calc©. Linear regression analysis was performed to find the factors that would be most influential to the hemoglobin level and development of anemia in the third trimester.

## 3. Results

**Figure 1:**
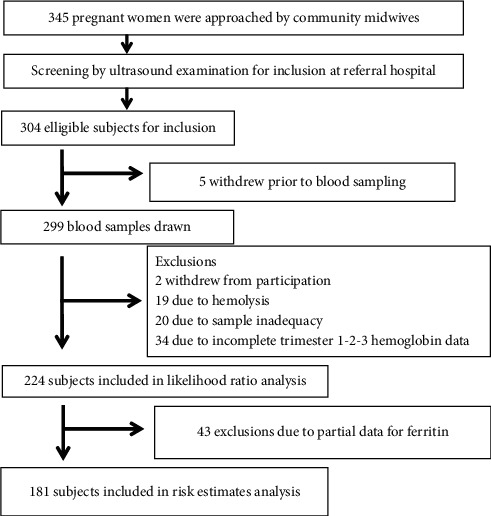
Inclusion and exclusion chart for data analysis.

The recruitment of pregnant women in the cohort is depicted in Figure 1.

Overall, there were 181 sets of data in the cohort which were eligible for analysis of anemia and the diagnostic study. The prevalence of anemia by consecutive trimesters was 8.48%, 14.29%, and 29.91%. Descriptive statistics of hemoglobin level in the third trimester are presented in Table 1.

The nonparametric correlation test showed that first trimester hemoglobin was significantly correlated with the second and third trimester hemoglobin (*r* = 0.596 and *r* = 0.565, *p* < 0.01). First trimester ferritin was significantly correlated with the first and third trimester hemoglobin (*r* = 0.182 and *r* = 0.321, *p* < 0.1). Hepcidin, sTfR, cholecalciferol, and calcitriol showed no correlation with hemoglobin at any trimester.

To find the best predictor of anemia, ROC analysis was performed on the five markers being evaluated. The results turned that ferritin was the only marker which fulfilled the criteria *p* < 0.05 (Table 2). All the other markers performed poorly on ROC analysis (data not shown). Table 2 presents the test details on ferritin.

Based on the new cutoff, the distribution of subjects in the groups changed as shown in Table 3.

Although the prevalence was still the same, the likelihood ratio (+) of the new cutoff point of ferritin was better, as shown in Table 4.

## 4. Discussion

Anemia among pregnant women has high prevalence worldwide, with possible serious fetal-maternal sequelae, such as low birth weight and preterm delivery [11]. This study was the continuation of the cohort which was previously reported [5], but with additional data from additional subjects and different purposes, new analyses were added. Some changes were observed in the prevalence of anemia in this study population. Although the curves and tables were not shown in this report, it was reconfirmed in the analysis that cholecalciferol and calcitriol in the first trimester had no correlation with anemia in the third trimester, and they came out poor in the ROC curve. The same thing was found for hepcidin and sTfR, which was unexpected as some experts consider that hepcidin and sTfR were superior to ferritin.

It was previously reported that anemia in the first trimester was 7.5% among the population in the cohort [5] which turned into 8.48% in this study. The new prevalence of anemia in the third trimester was 29.91%, which meant that there was 3.5 times increase from the prevalence in the first trimester. The prevalence of anemia in the third trimester in this study was still in the moderate level of public health significance [24]. The prevalence of anemia among pregnant women was 43% in Cambodia and 63% among pregnant women in Kolar, India [25, 26]. In Tasmania, the prevalence of anemia also increased by trimester, which were 13.4% in 14–25 weeks of gestations, 17.7% in 26–36 weeks, and 21.9% in 37–42 weeks of gestation [12]. However, the mean value for third trimester hemoglobin among women with anemia was lower in this study, at 9.96 gr/dl, which fell in the moderate category by the WHO [24]. The proportion of pregnant women with ferritin below 30 ng/ml was previously 24.9% [5]; in this final report, it slightly increased to 25.41%. The median for serum ferritin among pregnant women in Tasmania was 24.8 ng/ml, and 33.3% of the subjects fell below median value [12].

The area under curves in the ROC for hepcidin and sTfR were small, and they were of no significance. This had practically eliminated the two biomarkers as predictor for anemia. In a clinical trial, it was concluded that hepcidin-guided screen-and-treat approaches had no advantages over the WHO's recommended regimen in terms of adherence, side-effects, or safety outcomes [27]. In a review, it was concluded that hepcidin is a regulator of iron homeostasis and may be a useful biomarker to determine iron bioavailability in pregnancy, but there was no conclusive result for prediction of anemia [8]. Proper distinction between true iron deficiency anemia versus inflammation-mediated iron restriction during pregnancy may be helpful in clinical setting as iron may need to be prescribed appropriately [8]. In this study, first trimester hepcidin also performed poorly in prediction of anemia in the third trimester. The use of sTfR for prediction of anemia in several previous studies was conflicting [28–30]. But, it became clear that first trimester sTfR is not candidate for prediction in the third trimester.

In the applications for improving health care service, the findings of this study are very important. The new cutoff would give some benefit to reassure any pregnant woman with anemia and low ferritin level, to pay more attention on improving her health, and to avoid anemia in the third trimester and its detrimental consequences. There was a 4.8% increase in specificity (from 81.45 to 86.26). This means that an additional 5 out 100 pregnant women would be screened for treatment of restoring iron stores and that those without adequate management might fall worse.

Based on the correlation tests, only hemoglobin and ferritin in the first trimester had shown consistent result with third trimester hemoglobin level and the occurrence of anemia.

The serum ferritin level, known as one of many markers of iron stores, was below the two cutoff points evaluated in this study. It is of major concern as it resulted in approximately 3 times higher risk for anemia in the third trimester.

## 5. Conclusion

This cohort study provided evidence that the serum ferritin level in the first trimester (≤27.23 ng/ml) was the best marker to predict anemia in the third trimester. It will be valuable in targeting subjects for more rigorous approach for the prevention and treatment of anemia in pregnancy, especially in low-resource setting. The usefulness of ferritin as the marker for treatment of anemia in pregnancy would need a carefully designed randomized controlled trial.

## Figures and Tables

**Table 1:** Descriptive statistics of hemoglobin levels in the third trimester.

	Anemia	Non anemia	Total
	tm 3	tm 3	
*n* of subjects (%)	67 (29.91)	157 (70.09)	224 (100)
Hb mean (SD)	9.96 (0.88)	12.01 (0.77)	11.40 (1.24)
Hb median (IQR)	10.02 (1.20)	11.80 (1.05)	11.50 (1.47)
Hb min-max	7.3–10.90	11.0–14.7	7.3–14.70

tm3 = third trimester; *n* = number; Hb = hemoglobin; SD = standard deviation; IQR = interquartile range; Min = minimum value; max = maximum value. Kolmogorov-Smirnof test result *p* < 0.001; data were not normally distributed.

**Table 2:** Diagnostic study results on the ferritin level in the first trimester to predict anemia in the third trimester.

Variable classification	Ferritin and anemia
Sample size	181
Positive group	57 (31.49%)
Negative group	124 (68.51%)
Area under the ROC curve (AUC)	0.672
Standard error	0.0448
95% confidence interval	0.598 to 0.739
*z* statistic	3.829
Significance level *p* (area = 0.5)	0.0001
Youden index *J*	0.2840
Associated criterion	≤27.23
Sensitivity	42.11
Specificity	86.29
Likelihood ratio (+) (95% CI)	3.07 (1.8–5.3)
Likelihood ratio (−) (95% CI)	0.67 (0.5–0.8)

**Table 3:** Distribution of pregnant women with and without anemia based on two cutoff values of ferritin level.

Test results	Anemia (*n* (%))		Nonanemia (*n* (%))	
*New cutoff (ferritin ≤ 27.23 ng/ml)*				
Positive	24	(58.5)	17	(41.5)
Negative	33	(23.6)	107	(76.4)

*Pervious cutoff (ferritin < 30 ng/ml)*				
Positive	24	(52.2)	22	(47.8)
Negative	33	(24.4)	102	(75.6)

**Table 4:** Likelihood ratio and risk estimates of maternal ferritin level in the first trimester to predict anemia in the third trimester.

	Ferritin ≤ 27.23 ng/ml	Ferritin < 30 ng/ml
Number of subjects	181	181
Likelihood ratio (+) (95% CI)	3.07 (1.80–5.30)	2.27 (1.40–3.70)
Risk estimates (95% CI)	2.48 (1.67–3.68)	2.13 (1.42–3.20)

## Data Availability

The data used to support the study can be made available from the corresponding author upon reasonable request.
